# Vitamin A Uses Its Superpowers to Combat Vascular Malformations

**DOI:** 10.1016/j.jacbts.2026.101725

**Published:** 2026-09-26

**Authors:** Zaneta Markowska, Karen K. Hirschi

**Affiliations:** Department of Cell Biology and Developmental Genomics Center, Robert M. Berne Cardiovascular Research Center, University of Virginia School of Medicine, Charlottesville, Virginia, USA

**Keywords:** Glenn, hereditary hemorrhagic telangiectasia, pulmonary arteriovenous malformations, retinoic acid, single ventricle

**Figure:**
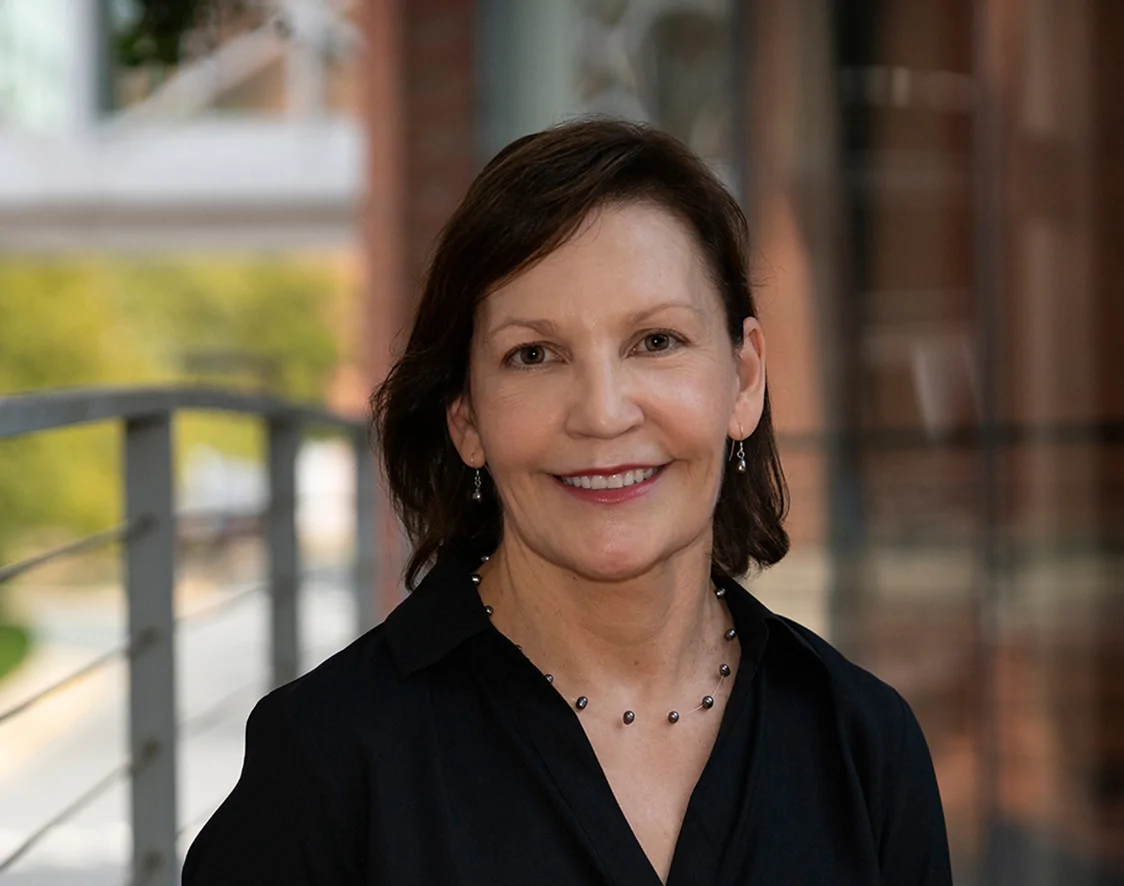

Blood vessels are composed of endothelial cells (ECs) that form the inner luminal layer and mural cells that form the surrounding vessel wall. During blood vessel development, ECs are specified to become distinct subtypes (arterial, venous, capillary) needed to form a systemic circulatory network. Disruptions in this process can lead to vascular malformations (VMs) of different types, depending on which vessels are affected; for example, capillary or venous malformations, or arterialvenous malformations (AVMs). Dysregulated blood vessels within VMs do not properly distribute nutrients and oxygen to tissues and are prone to rupture, thereby causing hypoxia and hemorrhage. VMs are particularly dangerous when they arise in the brain, where they can lead to neurologic damage and stroke,^1^ and in the lung, where they can cause bleeding and low blood oxygen levels (hypoxemia).^2^ Despite their clinical impact, the mechanisms driving the pathogenesis of VMs remain poorly understood.

Pulmonary vascular malformations (PVMs) are serious complications that develop in pediatric patients with single-ventricle congenital heart disease, who undergo life-saving surgical repair via the Glenn procedure. During this surgery, the superior vena cava is connected directly to the pulmonary arteries, allowing deoxygenated blood from the upper body to flow directly to the lungs without passing through the heart. Although this procedure improves systemic oxygenation, it also leads to the formation of abnormal blood vessels in the lungs, PVMs, that impair normal gas exchange, resulting in hypoxemia.^2^ Despite their serious clinical consequences, the mechanisms driving PVMs remain poorly understood.

Addressing this knowledge gap, Rousseau et al^3^ identified molecular drivers of the early stages of PVM initiation following the Glenn procedure at single-cell resolution in this issue of *JACC: Basic to Translational Science*. Using an established surgical model, they demonstrated an increased number of enlarged, dysplastic microvessels in post-Glenn lungs compared with lungs from sham control groups 3 weeks after surgery. Single-cell RNA sequencing of Glenn and control lungs revealed that the EC population therein was the most affected, with a significantly reduced proportion of lung capillary ECs. Differential gene expression analysis revealed that angiogenesis was the most dysregulated pathway among ECs, along with impaired inflammatory response pathways and reduced retinoic acid signaling.

To determine whether post-Glenn PVMs may be induced via mechanisms similar to hereditary hemorrhagic telangiectasia (HHT)-associated AVMs, Rousseau et al^3^ compared differential gene expression in ECs from Glenn lungs and mouse models of HHT. They identified common dysregulation of angiogenesis-related genes and reduced expression of genes involved in all-trans retinoic acid (ATRA) signaling. Consistent with these findings, spectral cytometry at 3 weeks post-Glenn confirmed a reduction in the number of retinoid^+^ lung ECs, as well as retinoid levels within these cells. The importance of this pathway was functionally validated in studies showing that mice fed a vitamin A–deficient diet developed an increased number of enlarged, dysplastic microvessels post-Glenn procedure compared with control groups, and this was reduced with supplemental vitamin A intake (Figure 1). Vitamin A deficiency was associated with upregulated phosphoinositide 3-kinase (PI3K)/Protein Kinase B signaling, and a vitamin A-excess diet was associated with its down-regulation, suggesting a potential mechanism through which dysregulated ATRA signaling induces PVMs and may similarly induce HHT-associated AVMs.

**Figure 1:**
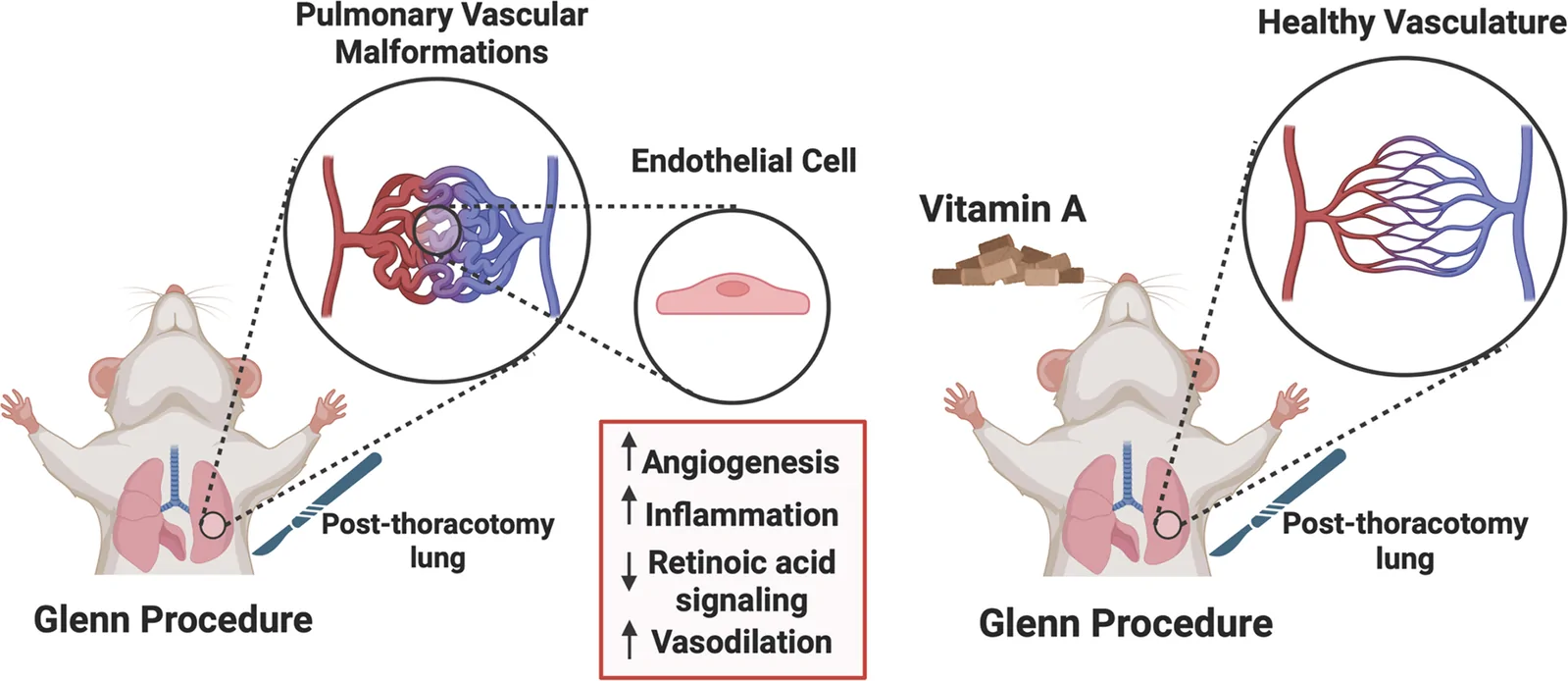
Vitamin A Prevents Pulmonary Vascular Malformations Following the Glenn Procedure (Rousseau et al^3^).

This study provides the first mechanistic insights into the initiation of PVMs following the Glenn procedure at single-cell resolution and implicates a novel pathway in the development of VMs. The authors further demonstrated that these changes occur predominantly in lung ECs, consistent with previous studies showing that pulmonary arterial ECs, but not smooth muscle cells, exhibit increased proliferation following the Glenn procedure.^4^ The identification of overlapping transcriptional changes between post-Glenn PVMs and HHT-associated AVMs raises an important translational possibility: Therapies currently used or under investigation for HHT could potentially be repurposed for patients who develop PVMs post-Glenn procedure. For example, antiangiogenic agents such as bevacizumab, which target vascular endothelial growth factor A signaling, have demonstrated clinical efficacy in patients with HHT and may warrant investigation as a potential therapeutic strategy for Glenn-induced PVMs.^5^

Importantly, Rousseau et al^3^ provided evidence for a protective role of ATRA signaling against post-Glenn PVMs through suppression of PI3K/AKT signaling. These findings raise the possibility that ATRA treatment before surgery may prevent PVMs, and restoring or enhancing ATRA signaling postsurgery could reduce/regress PVMs, and possibly HHT-associated AVMs. Notably, excessive PI3K signaling has previously been implicated in HHT-associated AVMs,^6^ suggesting a more broadly shared mechanistic link between impaired retinoid signaling and aberrant PI3K activation in VMs. Lastly, this study provides novel insights into the up-regulation of inflammatory signaling in pulmonary ECs following the Glenn procedure, which has not been previously reported. Inflammation has been implicated in AVM progression and rupture^7^ and proposed as a potential “second hit” that contributes to lesion formation in HHT.^8^ These findings suggest that inflammatory pathways may similarly contribute to PVM progression post-Glenn procedure. If confirmed, targeting inflammation, possibly with inhaled immunomodulatory therapies, could represent a novel therapeutic avenue for these patients.

Despite these important advances, several limitations should be considered. First, although the mouse model recapitulates key features of post-Glenn PVMs, differences between murine models and the human single-ventricle circulation may limit their clinical translation. Furthermore, the Glenn model alters both pulmonary blood flow and the source of circulating factors by excluding hepatic venous blood flow, making it difficult to distinguish effects mediated by altered hemodynamics from those caused by loss of hepatic-derived signals. Thus, upstream mechanisms responsible for reduced EC ATRA signaling remain to be elucidated. In particular, identifying hepatic vein–derived factors that are absent following the Glenn procedure and determining how they regulate EC retinoic acid signaling could provide critical insight into molecular mechanisms underlying PVM formation. Notably, rerouting hepatic blood flow to the lungs through the Fontan procedure has been reported to induce regression of severe pulmonary AVMs,^9^ further highlighting the potential importance of hepatic-derived factors in PVM pathogenesis.

Future studies are needed to determine whether restoration of retinoic acid signaling can reverse established PVMs or whether treatment must be initiated before malformations develop. It is also unknown whether transient vitamin A supplementation is sufficient to maintain protection against PVMs post-Glenn or whether continuous supplementation is required. The safety and potential toxicity of long-term retinoid treatment will also need to be carefully evaluated, given that this condition affects pediatric patients. Another important consideration is whether the dysregulated EC proliferation observed in post-Glenn PVMs is driven by impaired cell cycle control mechanisms. Notably, pharmacologic induction of cell cycle arrest using the Cyclin-Dependent Kinase 4/6 inhibitor palbociclib prevents AVM formation in a murine HHT model,^10^ suggesting that targeting aberrant EC cycle with such available drugs may be a promising strategy for Glenn-associated PVMs. Finally, determining whether impaired ATRA signaling is common across different types of VMs could establish the broader relevance of this pathway and its therapeutic targeting.

Overall, the identification of dysregulated ATRA signaling in the development of single-ventricle PVMs represents an important clinical contribution, providing new insights into underlying mechanisms and therapeutic targets for this poorly understood complication. Importantly, Rousseau et al^3^ also provide valuable, publicly accessible data sets defining the cellular populations involved in the development of single-ventricle PVMs that can serve as a resource for future investigations.

## Funding Support and Author Disclosures

The authors have reported that they have no relationships relevant to the contents of this paper to disclose.
